# Supplementary material for: Mechanism of IRSp53 inhibition by 14-3-3
Source: Nat Commun. 2019 Jan 29;10:483. doi: 10.1038/s41467-019-08317-8 (PMC6351565; doi:10.1038/s41467-019-08317-8)
Supplement: Supplementary file 1 — Supplementary Information [file 41467_2019_8317_MOESM1_ESM.pdf]

Supplementary information

Mechanism of IRSp53 inhibition by 14-3-3

David J. Kast[1,2] and Roberto Dominguez[1]

[1] Department of Physiology, Perelman School of Medicine, University of Pennsylvania, Philadelphia, PA 19104, USA

[2] Current address: Department of Cell Biology and Physiology, Washington University, St. Louis, MO 63110, USA

ORCID: DK, 0000-0002-1031-1211

ORCID: RD, 0000-0003-3186-5229

Correspondence and requests for materials should be addressed to R.D. (email: droberto@pennmedicine.upenn.edu)

Supplementary Table 1: Synthetic IRSp53 phospho-peptides

Peptide	Sequence
Site 1	^{319}AQPKSL(pS)PPQSQS^{331}
Site 2	^{333}LSDSYSN(pT)LPVRKS^{346}
Site 3	^{354}ATTENK(pT)LPRSSS^{366}
Site 4	^{360}TLPRSS(pS)MAAGLEK^{373}
Site 5	^{448}QQGKSS(pS)TGNLLD^{460}
Sites 1,2	^{319}AQPKSL(pS)PPQSQSKLSDSYSN(pT)LPVRKS^{346}
Sites 2,3	^{335}SDSYSN(pT)LPVRKSVTPKNSYATTENK(pT)LPRSSS^{366}
Sites 2,4	^{335}SDSYSN(pT)LPVRKSVTPKNSYATTENKTLPRSS(pS)MAAGLE^{372}

Supplementary Table 2: Crystallographic data and refinement statistics

Structure	Site 2 (pT340)	Site 3 (pT360)	Site 4 (pS366)	Sites 2,3 (pT340/pT360)	Sites 3,4 (pT340/pS366)
Data collection					
Beamline	NSLS X6A	MacCHESS A1	X8 Prospector	X8 Prospector	X8 Prospector
Wavelength (Å)	1.000	0.977	1.540	1.540	1.540
Space group	P 1	P 1	P 1	P 1	P 21 21 21
Cell *a*, *b*, *c* (Å)	60.0, 69.1, 84.6	59.4, 68.6, 84.8	59.9, 69.3, 84.7	61.1, 69.6, 150.5	69.9, 75.0, 100.2
Cell α, β, γ (°)	105.3, 95.7, 115.0	72.9, 85.6, 64.7	106.1, 95.6, 114.9	99.2, 93.0 116.0	90.0, 90.0, 90.0
Resolution (Å)	31.3–2.0 (2.06–2.0)[a]	21.9–2.3 (2.38–2.3)	29.4–2.35 (2.4–2.35)	43.0–2.8 (2.9–2.8)	60.0–2.9 (3.0–2.9)
R_{merge} (%)	4.2 (37.9)	8.7 (57.9)	5.8 (20.5)	14.2 (29.3)	27.8 (34.4)
$I / \sigma I$	21.8 (3.5)	12.5 (3.6)	20.0 (4.9)	20.5 (2.5)	17.0 (2.1)
No. unique reflections	76071 (6527)	49462 (4819)	45896 (4330)	51911 (5197)	12087 (1064)
Multiplicity	3.4 (2.7)	3.6 (3.2)	5.5 (2.7)	4.8 (2.1)	7.2 (1.9)
Completeness (%)	95.7 (92.0)	96.6 (94.3)	95.6 (90.4)	96.7 (95.7)	98.8 (91.4)
$CC_{1/2}$	0.999 (0.888)	0.996 (0.87)	0.998 (0.889)	0.975 (0.717)	0.887 (0.629)
Wilson B-factor (Å^2)	32.75	35.95	23.21	30.91	25.61
Refinement					
Resolution (Å)	31.3–2.0	21.9–2.3	29.4–2.35	43.0–2.8	60.0–2.9
No. reflections	76054	49428	45857	51490	12042
R_{work} (%) / R_{free} (%)	19.5 / 22.9	19.2 / 22.2	21.2 / 25.1	23.5 / 28.6	21.5 / 25.8
No. atoms					
Protein	7868	7763	7692	15360	3868
Ligands	92	111	70	212	50
Solvent (H_2O)	517	110	406	93	34
RMS deviations					
Bond lengths (Å)	0.003	0.002	0.002	0.005	0.004
Bond angles (°)	0.55	0.46	0.45	0.62	0.88
B-factors (Å^2)					
Protein	42.2	54.0	31.7	38.3	22.5
Ligand	52.4	60.9	40.6	46.5	34.5
Solvent	42.6	43.0	29.9	18.5	14.6
Ramachandran (%)					
Favored	99.1	98.6	98.9	97.3	98.3
Outliers	0.0	0.0	0.0	0.0	0.0
PDB code	6BCR	6BCY	6BD1	6BQT	6BD2

[a] Values in parentheses are for highest-resolution shell

Supplementary Table 3: Oligonucleotide sequences

Constructs	Sequence
IRSp53	
Primer: pEGFP-C1: NheI-IRSp53-FLAG (ΔGFP), Forward	GGTGCTAGCATGTCTCTGTCTCGCTCA GAGG
Primer: pEGFP-C1: IRSp53-FLAG-SalI (ΔGFP) 1 of 2, Reverse	CGTCATCGTCCTTGTAGTCTCCGDYKD DDCACTGTGCACACCAGCGTG
Primer: pEGFP-C1: IRSp53-FLAG-SalI (ΔGFP) 2 of 2, Reverse	ACCGTCGACCTACTTGTCGTCATCGTC CTTGTAGTCTCC
Primer: pEGFP-C1: IRSp53-STREP-SalI (ΔGFP) 1 of 2, Reverse	CTGAGGGTGGGACCAGCC CACTGTGGACACCAGCGTG
Primer: pEGFP-C1: IRSp53-STREP-SalI (ΔGFP) 2 of 2, Reverse	ACCGTCGACTCACTTCTCGAACTGAGG GTGGGACCAGCC
Primer: pIRES 1: NheI-IRSp53-FLAG, Forward	GGTGCTAGCATGTCTCTGTCTCGCTCA GAGG
Primer: pIRES 1: IRSp53-FLAG-EcoRI 1 of 2, Reverse	CGTCATCGTCCTTGTAGTCTCCGDYKD DDCACTGTGCACACCAGCGTG
Primer: pIRES 1: IRSp53-FLAG-EcoRI 2 of 2, Reverse	ACCGAATTC CTACTTGTCGTCATCGTCCTTGTAGTC
Primer: pIRES 2: SalI-IRSp53-STREP, Forward	GGTGTCGACATGTCTCTGTCTCGCTCA GAGG
Primer: pIRES 2: IRSp53-STREP-NotI 1 of 2, Reverse	CTGAGGGTGGGACCAGCC CACTGTGGACACCAGCGTG
Primer: pIRES 2: IRSp53-STREP-NotI 2 of 2, Reverse	ACCGCGGCCGC TCACTTCTCGAACTGAGGGTGG
Mutations of the phosphorylation sites of IRSp53	
QuickChange Primer: pEGFP Site 2 Mutation (T340A), Forward	CGACTCCTACTCCAACGCACTCCCCGT GCGCAAGAGC
QuickChange Primer: pEGFP Site 2 Mutation (T340A), Reverse	GCTCTTGCGCACGGGGAGTGCGTTGGA GTAGGAGTCG
QuickChange Primer: pEGFP Site 3 Mutation (T360A), Forward	GCCACCACCGAGAACAAGGCTCTGCCT CGCTCGAGC
QuickChange Primer: pEGFP Site 3 Mutation (T360A), Reverse	GCTCGAGCGAGGCAGAGCCTTGTTCTC GGTGGTGGC
QuickChange Primer: pEGFP Site 4 Mutation (S366A), Forward	GCCTCGCTCGAGCGCCATGGCAGCCG GCCTGG
QuickChange Primer: pEGFP Site 4 Mutation (S366A), Reverse	CCAGGCCGGCTGCCATGGCGCTCGAG CGAGGC
Primer pEGFP Site 5 Mutation (^{452}SSST^{455} to ^{452}AAAA^{455}), Forward	GCCGCCGCCGCCGGCAACCTCCTGGA CAAGG
Primer: pEGFP Site 5 Mutation (^{452}SSST^{455} to ^{452}AAAA^{455}), Reverse	GGCGGCGGCGGCCTTCCCTTGCTGCA GGCTC

Supplementary Table 3, continued: Oligonucleotide sequences

Constructs	Sequence
Eps8	
Primer: pEGFP-C1: NheI 2xFLAG Eps8 (ΔGFP) 1 of 3, Forward	CGACAAGGGAATGAATGGTCATATTTCT AATCATCCCAG
Primer: pEGFP-C1: NheI 2xFLAG Eps8 (ΔGFP) 2 of 3, Forward	GGACGATGACGACAAGGGAATGAATGG TCATATTTC
Primer: pEGFP-C1: NheI 2xFLAG Eps8 (ΔGFP) 1 of 3, Forward	ATGGGAGACTACAAGGACGATGACGAC AAGGGA
Primer: pEGFP-C1: 2xFLAG Eps8 (ΔGFP) SalI, Reverse	GGTGTCGACTTAGTGACTGCTTCCTTCA TCAAAAGATT
14-3-3	
Primer: pTYB11: SapI-14-3-3, Forward	GGTTGCTCTTCCAACATGGAGAAGACT GAGCTGATCCAG
Primer: pTYB11: 14-3-3-PstI, Reverse	GGTCTGCAGTCATTAGTTTTCAGCCCCT TCTGCC
Primer: pMal-c2e: EcoRI-TEV-14-3-3 1 of 2 Forward	CCTGTATTTTCAGTCTATGGAGAAGACT GAGCTGATCCAG
Primer: pMal-c2e: EcoRI-TEV-14-3-3 2 of 2, Forward	GGTGAATTCGAAAACCTGTATTTTCAGT CTATGGAGAAGACTGA
Primer: pMal-c2e: 14-3-3-SalI, Reverse	GGTGTCGACTTAGTTTTCAGCCCCTTCT GCC

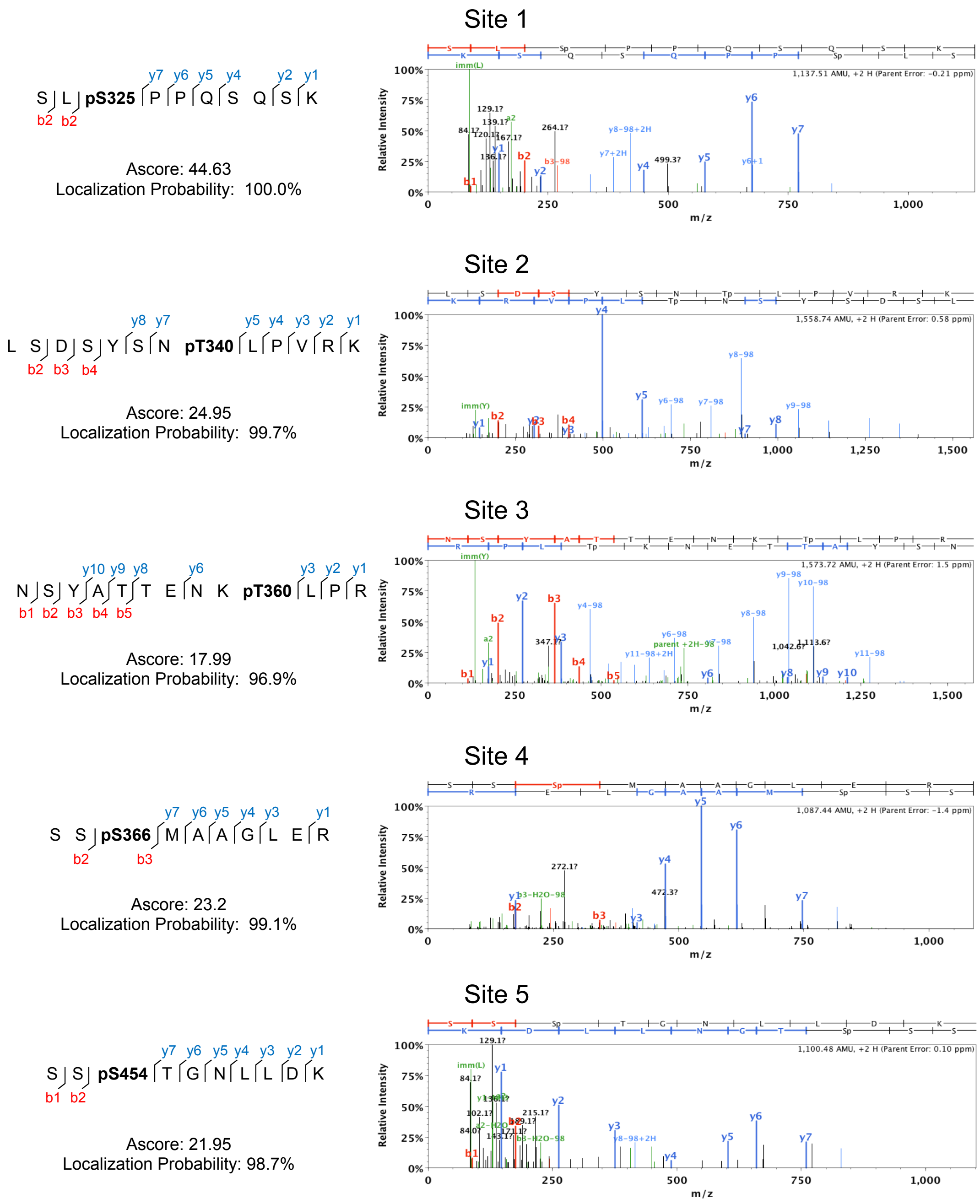

Supplementary Fig. 1: Representative MS/MS spectra of the five reproducibly phosphorylation sites found in pIRSp53. The phosphorylation site (bold characters), y- (blue) and b-ion (red) series are indicated on the peptide sequences. The localization probabilities of the assigned phosphorylation sites were validated using the Ascore algorithm (see Methods).

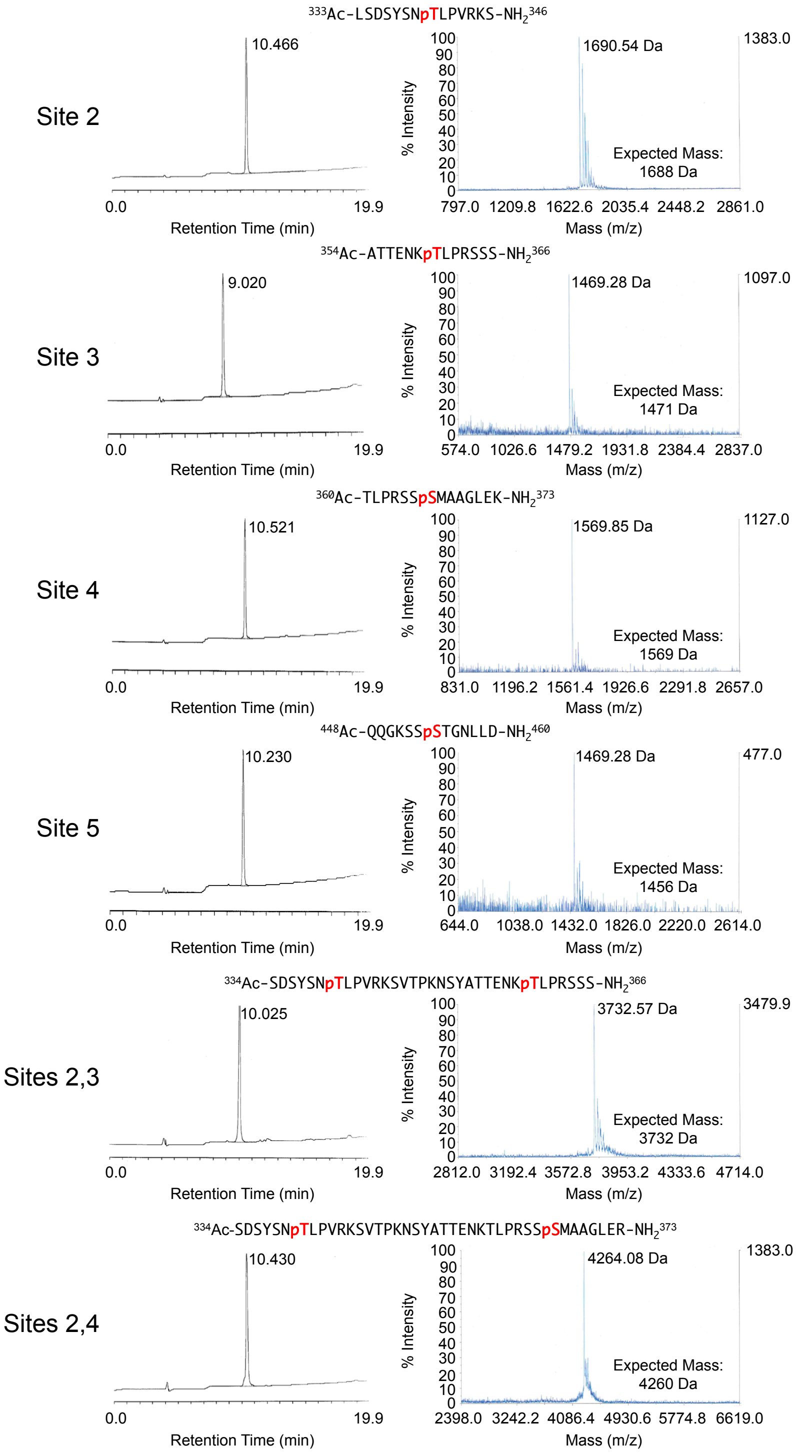

Supplementary Fig. 2: Synthetic singly- and doubly-phosphorylated peptides of IRSp53. The purity of each peptide was confirmed using HPLC (left panels) and their masses were validated using MALDI-TOF mass spectrometry (right panels). In each case the measured mass was nearly identical to the theoretical mass of the phosphorylated peptides.

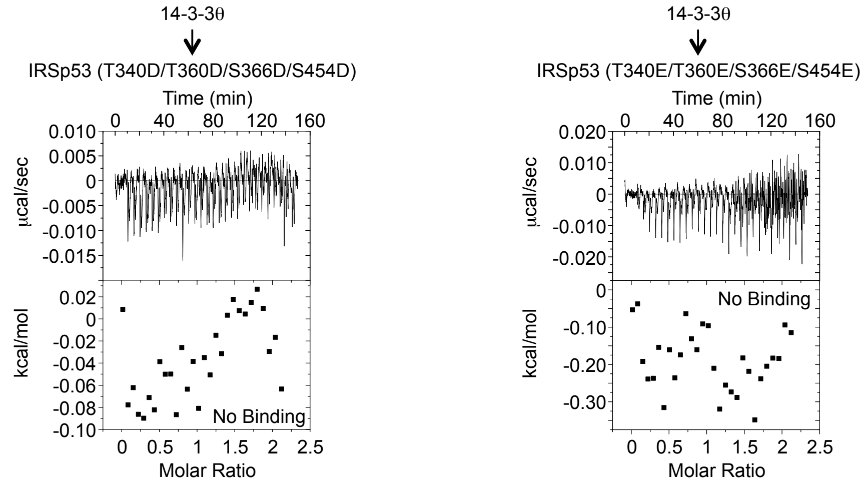

Supplementary Fig. 3: 14-3-3 does not bind to phosphomimetic mutants of IRSp53. ITC titrations of 200 μM 14-3-3θ into 20 μM *E. coli*-expressed IRSp53 carrying S/T to D or E mutations of phosphorylation sites 2, 3, 4 and 5 (as indicated). Experiments were performed at 20°C. Systematic heat differences were not observed (indicating lack of binding) and thus the titrations could not be fitted.

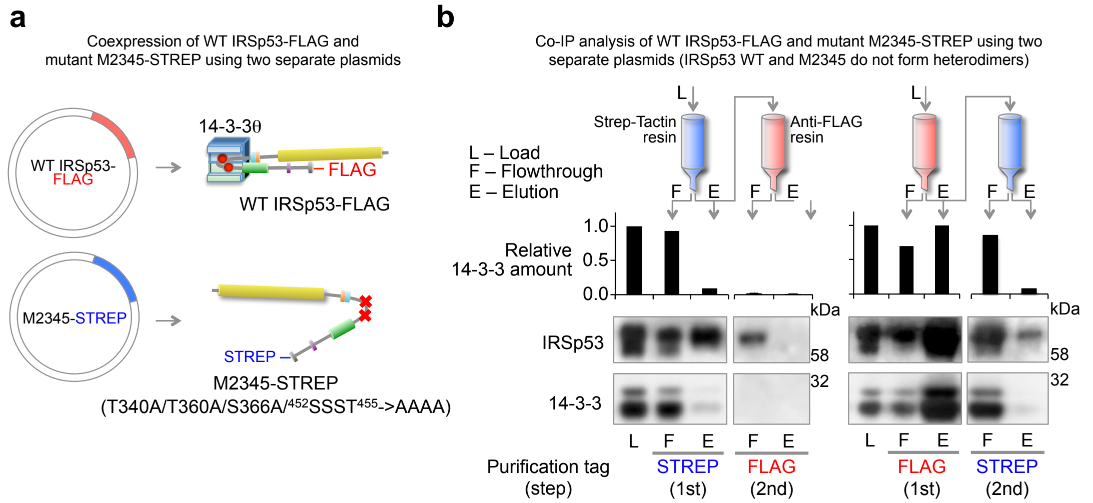

Supplementary Fig. 4: WT/mutant IRSp53 heterodimers do not form when co-expressed using two different plasmids. **a** Illustration of the unsuccessful strategy used to co-express WT/mutant IRSp53 heterodimers in HEK293T cells using two separate plasmids and two different affinity tags (IRSp53-FLAG and M2345-STREP). The IRSp53 mutant (M2345) carries mutations at site 2 (T340A), site 3 (T360A), site 4 (S366A) and site 5 (S452A-S453A-S454A-T455A). **b** While this strategy did not produce heterodimers, the results were nonetheless informative since endogenous 14-3-3 only bound to WT IRSp53, as illustrated by the amounts of 14-3-3 that coimmunoprecipitates with IRSp53 after affinity purification through two consecutive affinity columns, independent of the order of the purification. The results also demonstrate that IRSp53 does not form heterodimers when expressed from two separate plasmids, and thus IRSp53 dimerization must occur during translation and once formed the dimers never dissociate.

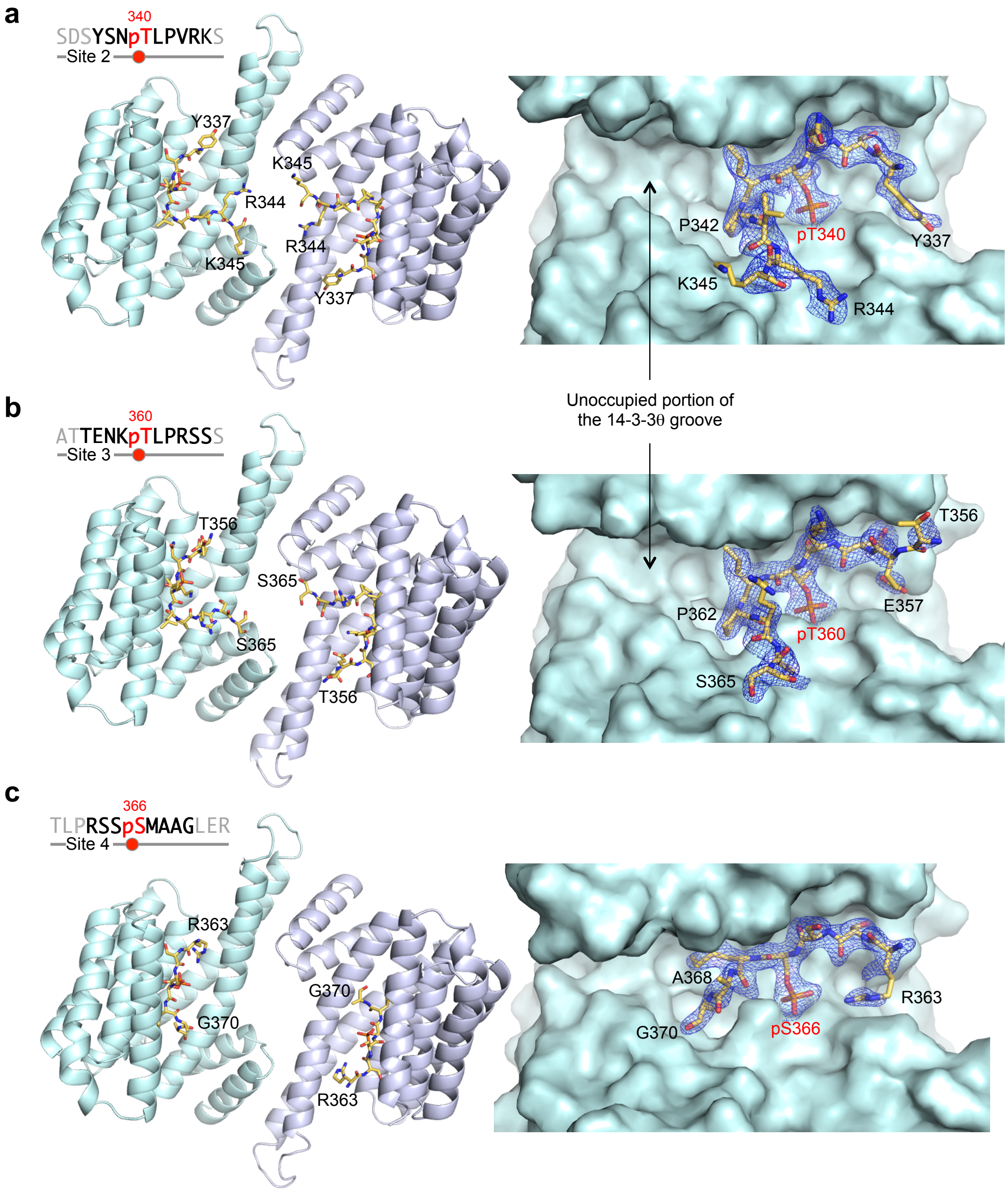

Supplementary Fig. 5: Structures of complexes of 14-3-3θ with singly-phosphorylated IRSp53 peptides. **a-c** Complexes of 14-3-3θ with the Site 2, Site 3 and Site 4 peptides of IRSp53, showing the overall structure (left) and close-ups of one of the binding pockets of 14-3-3θ (right), including the experimentally observed regions of the peptides and the corresponding $2F_o$-F_c electron density maps contoured at 1σ. Note that in the case of singly-phosphorylated peptides, both pockets of 14-3-3θ bind the same peptide in a pseudo-symmetric manner. In the sequence diagrams shown, the regions of the peptides observed in the structures are highlighted bold, the phosphorylation sites are highlighted red, and the portions of the peptide unresolved in the structures are shown in gray.

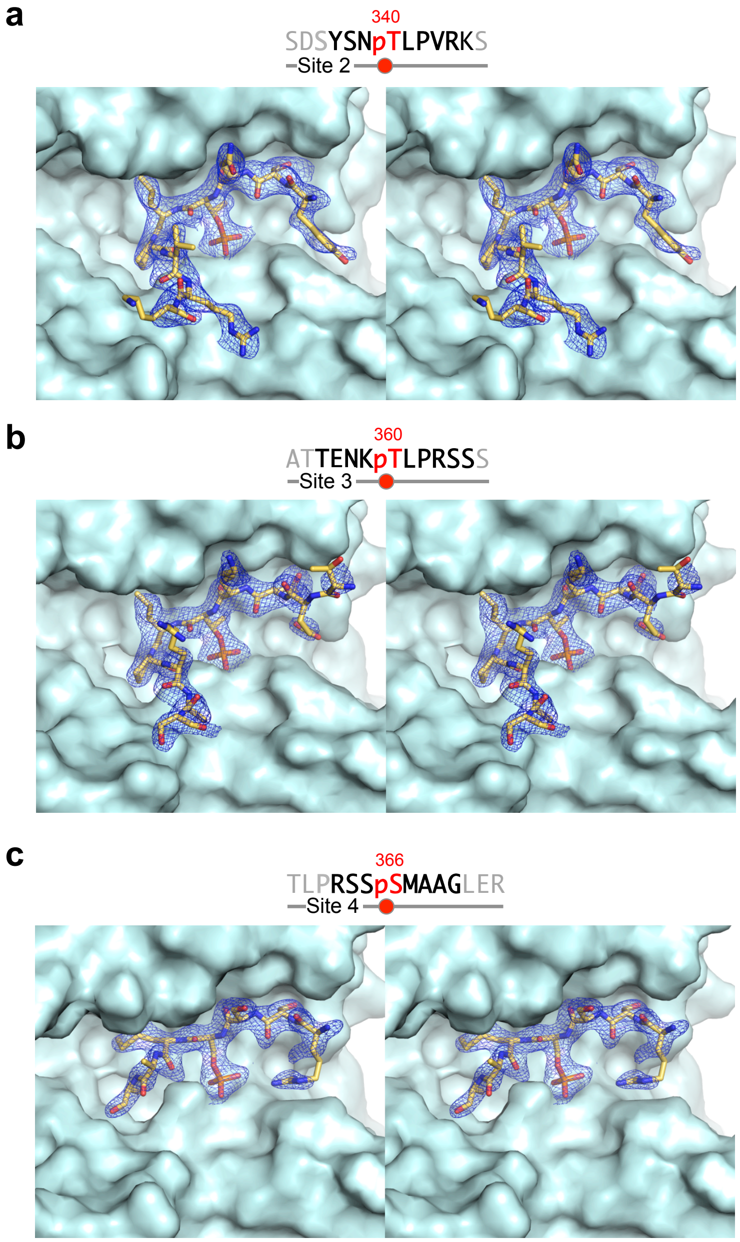

Supplementary Fig. 6: Stereo images of the peptide-binding pocket of 14-3-3θ showing the electron density map for the singly-phosphorylated peptides. **a-c** Complexes of 14-3-3θ with the Site 2, Site 3 and Site 4 peptides of IRSp53, showing the $2F_o$-F_c electron density maps contoured at 1σ. In the sequence diagrams, the regions of the peptides observed in the structures are highlighted bold, the phosphorylation sites are highlighted red, and the portions of the peptide unresolved in the electron density maps are shown in gray.

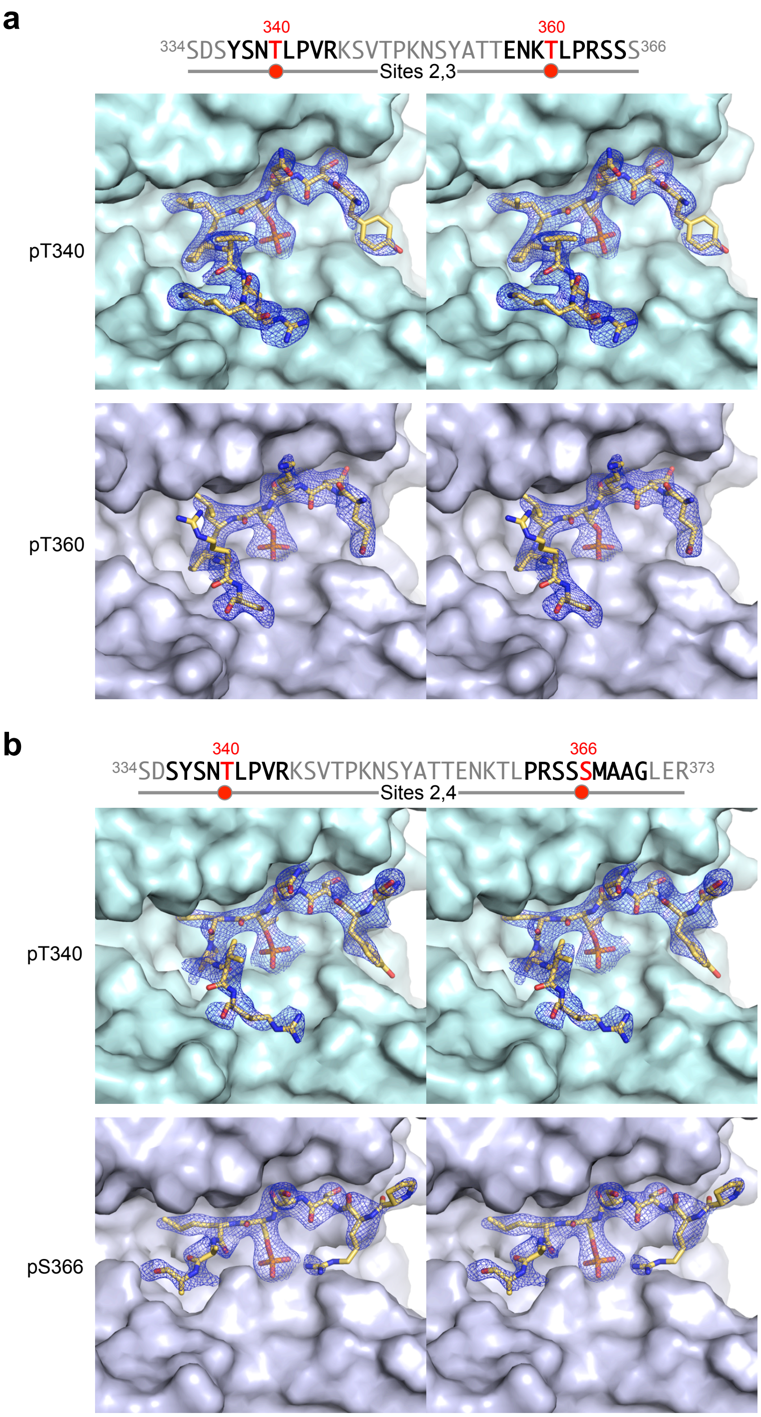

Supplementary Fig. 7: Stereo images of the peptide-binding pockets of 14-3-3θ showing the electron density map for the doubly-phosphorylated peptides. **a-b** Complexes of 14-3-3θ with the Sites 2,3 and Sites 2,4 peptides of IRSp53, showing the $2F_o$-F_c electron density maps contoured at 1σ. In the sequence diagrams, the regions of the peptides observed in the structures are highlighted bold, the phosphorylation sites are highlighted red, and the portions of the peptide unresolved in the electron density maps are shown in gray.

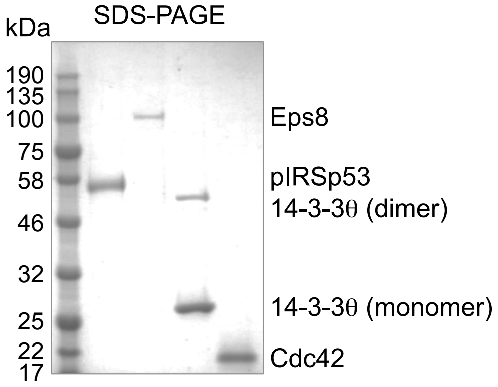

Supplementary Fig. 8: Proteins used in this study. Coomassie-stained SDS-PAGE illustrating the purity of several of the proteins used in this study: pIRSp53, Eps8, 14-3-3θ and Cdc42 (G12V). pIRSp53 and Eps8 were expressed in mammalian cells, whereas 14-3-3θ and Cdc42 were expressed in *E. coli*. (see Methods).

Fig. 1a-c

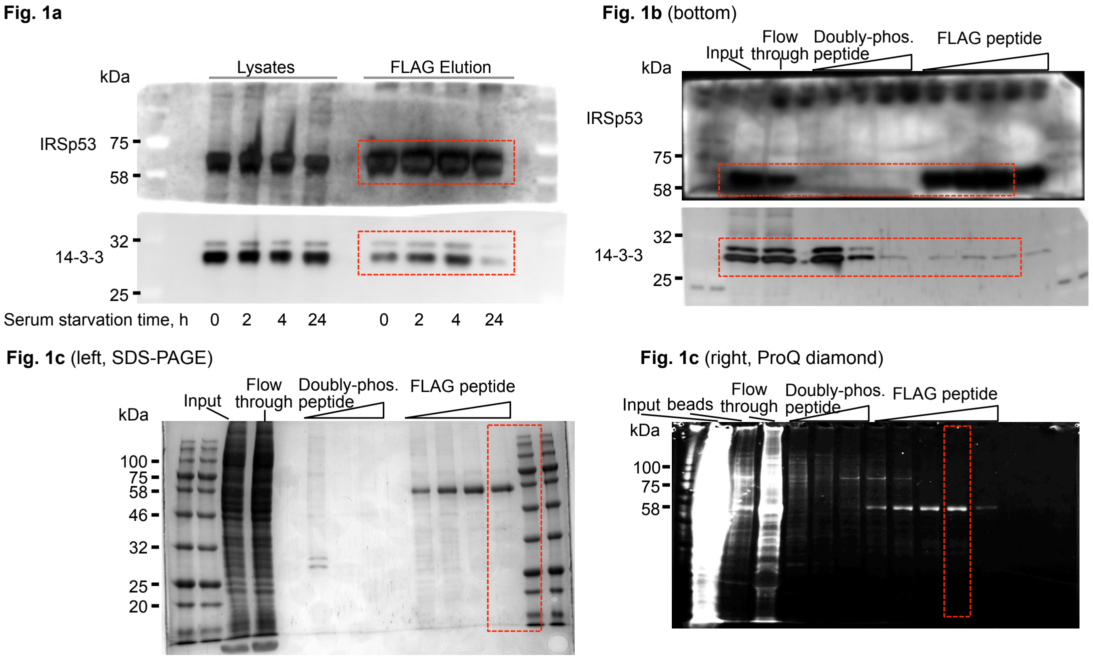

Fig. 2f

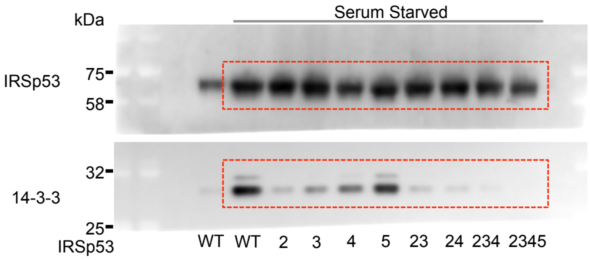

Fig. 3a and 3b

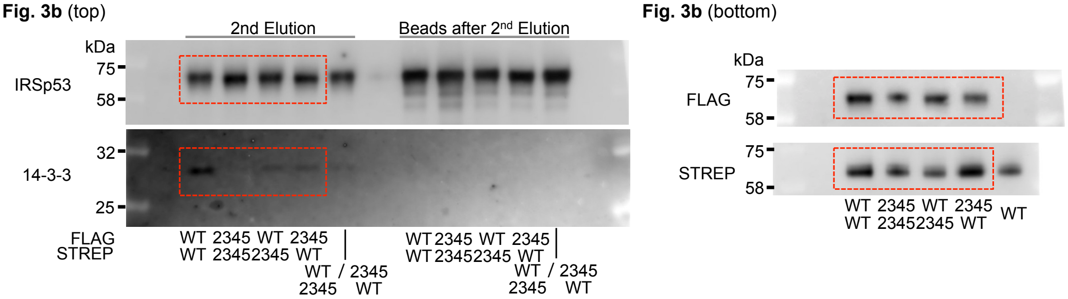

Supplementary Fig. 9: Original polyacrylamide gels and western blots. Red boxes mark the regions of the polyacrylamide gels or western blots that were used in the indicated Figs and Supplementary Figures.

Fig. 5f

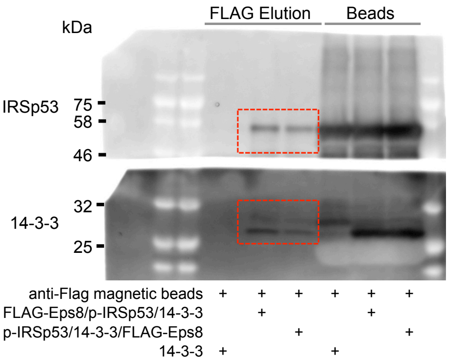

Fig. 6a

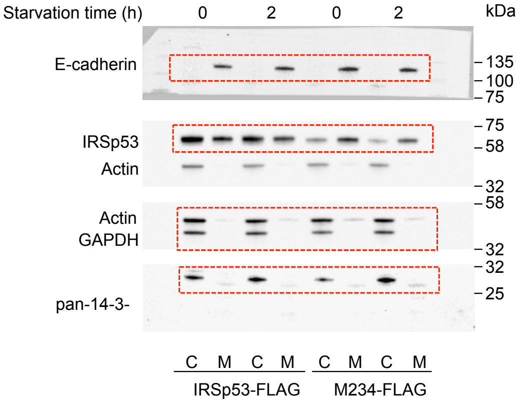

Supplementary Fig. 3f

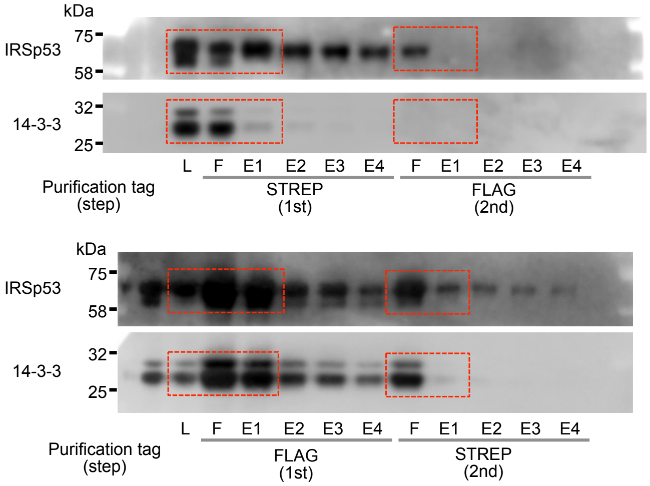

Supplementary Fig. 2c

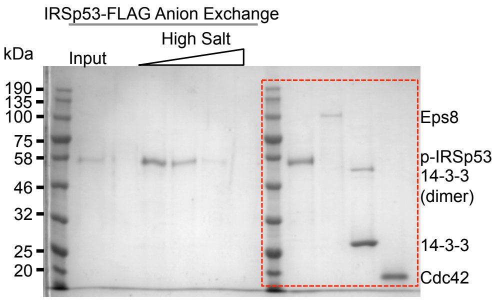

Supplementary Fig. 9, continued: Original polyacrylamide gels and western blots.
