# Supplementary material for: Mechanism of IRSp53 inhibition by 14-3-3
Source: Nat Commun. 2019 Jan 29;10:483. doi: 10.1038/s41467-019-08317-8 (PMC6351565; doi:10.1038/s41467-019-08317-8)
Supplement: Supplementary file 3 — Description of Additional Supplementary Files [file 41467_2019_8317_MOESM3_ESM.docx]

**Description of Additional Supplementary Files**

**File Name:** Supplementary Data 1

**Description:** Proteomic analysis of all identified sites of posttranslational modification in IRSp53 purified from serum starved mammalian cells.
