# Supplementary material for: Mechanism of IRSp53 inhibition by 14-3-3
Source: Nat Commun. 2019 Jan 29;10:483. doi: 10.1038/s41467-019-08317-8 (PMC6351565; doi:10.1038/s41467-019-08317-8)
Supplement: Supplementary file 5 — Reporting Summary [file 41467_2019_8317_MOESM5_ESM.pdf]

nature research

Corresponding author(s): Roberto Dominguez

Last updated by author(s): Dec 10, 2018

# Reporting Summary

Nature Research wishes to improve the reproducibility of the work that we publish. This form provides structure for consistency and transparency in reporting. For further information on Nature Research policies, see Authors & Referees and the Editorial Policy Checklist.

## Statistics

For all statistical analyses, confirm that the following items are present in the figure legend, table legend, main text, or Methods section.

| n/a | Confirmed | |
|---|---|---|
| ☐ | ☒ | The exact sample size (*n*) for each experimental group/condition, given as a discrete number and unit of measurement |
| ☐ | ☒ | A statement on whether measurements were taken from distinct samples or whether the same sample was measured repeatedly |
| ☐ | ☒ | The statistical test(s) used AND whether they are one- or two-sided<br>*Only common tests should be described solely by name; describe more complex techniques in the Methods section.* |
| ☐ | ☒ | A description of all covariates tested |
| ☐ | ☒ | A description of any assumptions or corrections, such as tests of normality and adjustment for multiple comparisons |
| ☐ | ☒ | A full description of the statistical parameters including central tendency (e.g. means) or other basic estimates (e.g. regression coefficient) AND variation (e.g. standard deviation) or associated estimates of uncertainty (e.g. confidence intervals) |
| ☐ | ☒ | For null hypothesis testing, the test statistic (e.g. *F*, *t*, *r*) with confidence intervals, effect sizes, degrees of freedom and *P* value noted<br>*Give P values as exact values whenever suitable.* |
| ☒ | ☐ | For Bayesian analysis, information on the choice of priors and Markov chain Monte Carlo settings |
| ☒ | ☐ | For hierarchical and complex designs, identification of the appropriate level for tests and full reporting of outcomes |
| ☒ | ☐ | Estimates of effect sizes (e.g. Cohen's *d*, Pearson's *r*), indicating how they were calculated |

*Our web collection on statistics for biologists contains articles on many of the points above.*

## Software and code

Policy information about availability of computer code

| | |
|---|---|
| Data collection | ITC data was acquired using Origin (Origin Labs; v7.0). Crystallography data was processed using HKL2000 (HKL Research) and SAINT (Bruker, v8.34A). Multi-angle light scattering data was collected using Astra (Wyatt Technology). |
| Data analysis | Crystallography data was analyzed using Phenix (www.phenix-online.org; v1.12) and Coot (www2.mrc-lmb.cam.ac.uk; v0.81). Densitometry analysis was performed with Fiji/ImageJ (https://fiji.sc; v2.0.0). ITC data was fitted using Origin (Origin Labs; v7.0). Phosphoproteomics data was analyzed using Scaffold (Proteome Software Inc.; v4.6.1), Scaffold PTM (Proteome Software Inc.; v3.2.0). Statistical analysis was performed using Prism (GraphPad Software, Inc., v8.0.1). Curve fitting of FRET data was performed using IGOR Pro (Wavemetrics; v7.0.0). Multi-Angle light scattering analysis was performed using Astra (Wyatt Technology). |

For manuscripts utilizing custom algorithms or software that are central to the research but not yet described in published literature, software must be made available to editors/reviewers. We strongly encourage code deposition in a community repository (e.g. GitHub). See the Nature Research guidelines for submitting code & software for further information.

## Data

Policy information about availability of data

All manuscripts must include a data availability statement. This statement should provide the following information, where applicable:

- Accession codes, unique identifiers, or web links for publicly available datasets
- A list of figures that have associated raw data
- A description of any restrictions on data availability

The coordinates and structure factors of the five structures of complexes of 14-3-3θ with singly- and doubly-phosphorylated IRSp53 peptides have been deposited in the Protein Data Bank (PDB) with accession codes 6BCR, 6BCY, 6BQT, 6BD2, and 6BD2.

# Field-specific reporting

Please select the one below that is the best fit for your research. If you are not sure, read the appropriate sections before making your selection.

☒ Life sciences ☐ Behavioural & social sciences ☐ Ecological, evolutionary & environmental sciences

For a reference copy of the document with all sections, see nature.com/documents/nr-reporting-summary-flat.pdf

# Life sciences study design

All studies must disclose on these points even when the disclosure is negative.

| | |
|---|---|
| Sample size | No sample-size calculations were performed. Sample size was determined to be adequate based on the magnitude and consistency of measurable differences between groups. |
| Data exclusions | No data was excluded from analysis. |
| Replication | In most cases all findings were reproduced at least 3 times. |
| Randomization | No randomization of any experiments. When possible, all experiments were paired with negative and positive controls. |
| Blinding | Investigators were not blinded to any of the experiments. Data reported for phosphoproteomics, co-ip, ITC, FRET, and crystallography experiments are not subjective and are based on quantitative methods. |

# Reporting for specific materials, systems and methods

We require information from authors about some types of materials, experimental systems and methods used in many studies. Here, indicate whether each material, system or method listed is relevant to your study. If you are not sure if a list item applies to your research, read the appropriate section before selecting a response.

## Materials & experimental systems

| n/a | Involved in the study | |
|---|---|---|
| ☐ | ☒ | Antibodies |
| ☐ | ☒ | Eukaryotic cell lines |
| ☐ | ☐ | Palaeontology |
| ☐ | ☐ | Animals and other organisms |
| ☐ | ☐ | Human research participants |
| ☐ | ☐ | Clinical data |

## Methods

| n/a | Involved in the study | |
|---|---|---|
| ☐ | ☐ | ChIP-seq |
| ☐ | ☐ | Flow cytometry |
| ☐ | ☐ | MRI-based neuroimaging |

## Antibodies

| | |
|---|---|
| Antibodies used | Mouse monoclonal anti-IRSp53 (Santa Cruz; sc-136470), rabbit polyclonal anti-pan-14-3-3 (Santa Cruz; sc-629), mouse monoclonal anti-β-actin (Santa Cruz; sc-47778), mouse monoclonal anti-GAPDH (Santa Cruz; sc-32233), rabbit polyclonal anti-FLAG-OctA (Santa Cruz; sc-807), mouse monoclonal anti-E-cadherin antibody (BD Biosciences; 610181), HRP-linked anti-mouse (Cell Signaling Technologies; 7076S), anti-rabbit IgG (Cell Signaling Technologies; 7074S) and HRP-conjugated Streptavidin (Thermo Fisher Scientific; N100) |
| Validation | Anti-IRSp53 (https://datasheets.scbt.com/sc-136470.pdf) was validated for western blotting (WB) of human, mouse, rat and canine IRSp53. Anti-pan-14-3-3was validated for mouse, rat, human and avian origin by WB, immunoprecipitation (IP), immunofluorescence (IF), immunohistochemistry (IHC(P)), Flow Cytometry (FCM) and ELISA; also reactive with additional species, including and equine, canine, bovine, porcine and avian. Anti-β-actin (https://datasheets.scbt.com/sc-47778.pdf) was validated to recognize β-Actin of mouse, rat, human, avian, bovine, canine, porcine, rabbit, Dictyostelium discoideum and Physarum polycephalum origin by WB, IP, IF, IHC(P) and ELISA; it may also cross-react with all six known isoforms of Actin in higher vertebrates. Anti-GAPDH (https://datasheets.scbt.com/sc-32233.pdf) was validated for detection of GAPDH of mouse, rat, human, rabbit by WB, IP and IF. Anti-FLAG-OctA was validated for detection of FLAG®-tagged fusion proteins by WB, IP, IF, IHC(P) and ELISA. The anti-E-cadherin antibody was validated for use in western blotting (www.bdbiosciences.com/ds/pm/tds/610181.pdf) shown to have reactivity for Human, mouse, rat and dog E-cadherin and some cross-reactivity with P-Cadherin. |

## Eukaryotic cell lines

Policy information about cell lines

| | |
|---|---|
| Cell line source(s) | HEK 293T/17 (ATCC; CRL-11268; ) |

| | |
|---|---|
| Authentication | Authenicated HEK 293T/17 cells were puchased directly from ATCC |
| Mycoplasma contamination | Cell lines have not been tested since the time of purchase. |
| Commonly misidentified lines (See ICLAC register) | *Name any commonly misidentified cell lines used in the study and provide a rationale for their use.* |
