# Supplementary material for: Mechanism of IRSp53 inhibition by 14-3-3
Source: Nat Commun. 2019 Jan 29;10:483. doi: 10.1038/s41467-019-08317-8 (PMC6351565; doi:10.1038/s41467-019-08317-8)
Supplement: Supplementary file 2 — Peer Review File [file 41467_2019_8317_MOESM2_ESM.pdf]

Reviewers' comments:

Reviewer #1 (Remarks to the Author):

The manuscript ‘Mechanism of IRSp53 inhibition by 14-3-3’ by Kast and Dominguez reports a study to decipher the structural and mechanistic features of the phosphoresidue-binding adaptor protein 14-3-3 to the I-BAR domain protein ITSp53 that plays an essential role in filopodia dynamics.

IRSp53 couples GTPase signaling with structural remodeling of the cytoskeleton and the membrane. In this role, IRSp53 is implicated in a number of normal development processes, but also in the pathology of for example cancer and neurological disorders. This fact is briefly mentioned in the introduction, maybe here (or later in the discussion) it would be helpful for the reader not accustomed to the role of IRSp53 to discuss the consequences of the negative influence of 14-3-3 on IRSp53. What might be the consequence of the here described regulation of 14-3-3 on IRSp53? What is the relevance of these findings for tumorigenesis, tumor progression, or metastasis? What does the community know about the possible effects in neurological disorders?

Back to the manuscript and the presented results, this study aimed at answering the following questions:

1) What are the phosphorylation sites in IRSp53 that mediate binding to 14-3-3 proteins?

2) What might be the protein kinases responsible for phosphorylating these 14-3-3 binding sites?

3) How do the multiple phosphorylation sites work together to convey 14-3-3 binding?

4) What is the stoichiometry of the interaction?

5) What is the effect of 14-3-3 binding on the overall conformation and activity state of IRSp53?

6) What is the relationship between 14-3-3 binding and the interactions of other partner proteins binding to IRSp53?

These questions were answered in the manuscript by a series of very elegant experiments. First the authors purified phosphorylated and 14-3-3 binding competent IRSp53 from HEK293 cells, using both a FLAG-tag and 14-3-3 affinity chromatography in a sequential manner to obtain pure and intact IRSp53, as can be judged from Figure 1c. (I wonder if the authors’ did try to reconstitute this protein with heterologously in E. coli expressed 14-3-3 for co-crystallization, SAXS or cryo-EM…) For this purification it was helpful that the authors found out that previously reported 14-3-3 binding site in IRSp53 could be phosphorylated by AMPK, which can be induced by staving the cells prior to purifying the protein.

Next, further 14-3-3 interaction sites were predicted by in-silico methods and the phosphorylation status of these sites were tested using phosphoproteomics on purified IRSp53, revealing 5 sites were

indeed phosphorylated in cells. These putative 14-3-3 binding sites were then synthesized as either mono- or di-phospho peptides and tested via ITC for their ability to bind to 14-3-3. The advantage of ITC is that it not only delivers a Kd of the interaction but also the molar ratio and thermodynamic parameters, confirming the validity of 4 of the 5 predicted 14-3-3 binding sites, a result further corroborated by site specific mutation of these sites in full-length IRSp53 and 14-3-3 pulldowns from cells.

In the following, the expression of heterodimers of IRSp53 with one protomer no longer able to bind 14-3-3, resulted in the reduction of IRSp53 bound 14-3-3 by 50%, hinting at a binding stoichiometry of one IRSp53 monomer binding one 14-3-3 dimer, thus a 2:4 (IRSp53:14-3-3) complex. This result was strengthen by experiments using a FRET assay.

The next step in elucidating structural details of the 14-3-3/IRSp53 interaction was the elucidation of the crystal structures of 3 binding motifs in complex with 14-3-3 as singly phosphorylated peptides and two combinations (2,4 and 2,3) of these sites as doubly-phosphorylated synthetic peptides. Here, it is of importance that the simultaneous binding of two sites within one 14-3-3 dimer could be shown and that the structures of the single-site phosphorylation with 14-3-3 showed very similar binding architectures as the same sites in the doubly-phophorylated constructs. Maybe it would be a good idea to prepare a figure for the SI with a structural alignment of the 340, 360 and 366 sites between the mono- and the double-phosphopeptide structures reporting also the rsmd.

Finally, by employing a FRET sensor construct, the effect of 14-3-3 binding on the overall structure of IRSp53 and the influence of other partner proteins like Cdc42C andEps8 were analyzed.

All in all, this is a very important study reporting data of highest technical value that explain how the important protein IRSp53 is regulated by 14-3-3 proteins. The only thing that might be added is a short discussion if and how these structural and mechanistic insights could be useful for example for potential drug discovery or chemical biology probe design.

Minor remarks:

Page 10: '…we expressed in HEK293T cell a modified…' should be '…we expressed in HEK293T cells a modified…'

Page 10: '…this mutant fails to coimmunoprecipitation 14-3-3 from cells...' should be '…this mutant fails to coimmunoprecipitate 14-3-3 from cells…'

Reviewer #2 (Remarks to the Author):

This manuscript by Kast and Dominguez describes detailed studies of IRSp53 and 14-3-3 protein binding. The work reported in this manuscript is highly mechanistic and appears to be performed very well. I only have a few points of questions regarding this work. Overall, I think the work is very much straightforward and easy to understand. I thought the discussion section could be more detailed in terms of discussing the proposed model, as opposed to talking about the pros and cons of various different online servers for protein-binding predictions. This part felt like it came out of nowhere.

Dye-labeling is performed in a way to produce a mixture with variable labeling orientations. As such, it should be stated that experiments were performed using a single preparation of the labeled probes to make sure that assays were performed using a consistent/identical batch of probes.

Further increase in FRET, though small, was noted when Cdc42 was titrated into 14-3-3/pIRSP53fs, as in Fig.5c. Figure 5e shows a clear case of where there's no additional change in FRET upon titration of Eps8 (a complete flat line), but Fig. 5c shows a downward sloping curve as a function of increasing Cdc42 concentration. This needs to be explained. In the text, it simply says there was no effect from titrating in additional Cdc42 which is clearly not what the data suggests.

In Figure 5g (right side blue colored plot), titration w/ Eps8 is shown in the figure panel, but the text states that this is titrated with 14-3-3 protein instead.

Is Rac1 able to compete for activation identically to Cdc42? The main abstract/intro discusses Cdc42 and filopodia. Rac1-IRSP53 and signaling through WAVE-Arp2/3 should be discussed to give broader perspective.

I recommend professional editorial service: writing is not clear in a number of places (does not flow). I had no idea what "servers" meant in the discussion, it just came out of the blue until I realized later that this is something about a web-based server platform… also some typos were noted.

Reviewer #3 (Remarks to the Author):

Comments for Kast et al

This paper describes interesting structure function relationship of the phosphorylation and the Cdc42 binding to IRSp53, a well-known I-BAR domain-containing protein for membrane protrusions. The AMPK induced phosphorylation of IRSp53 appeared to induce the binding of 14-3-3, and thereby inhibiting the IRSp53 by the clamp-like binding. The model is quite interesting, but I would like to point several issues that are unclear in the manuscript.

1. The responsible kinase is AMPK and some other kinases. The previous literatures said GSK3beta or some related kinase might phosphorylate IRSp53. The AMPK-induced phosphorylation and the suppression of the IRSp53 for the membrane binding looks to be reasonable because AMPK is activated upon serum starvation, which normally inactivates Cdc42. But how do the other kinases fit to the inhibition of IRSp53?

2. The output of the phosphorylation-induced 14-3-3 binding appears to be the suppression of the membrane binding. The change in the membrane binding should be shown experimentally in the presence of 14-3-3.

3. The effect of 14-3-3 on cellular function of IRSp53, filopodia, might be better to be confirmed in your system.

Minor point

On page 2, BAIA2 should be BAIAP2.

Point-by-point response to the Reviewers' comments:

Reviewer #1 (Remarks to the Author):

The manuscript 'Mechanism of IRSp53 inhibition by 14-3-3' by Kast and Dominguez reports a study to decipher the structural and mechanistic features of the phosphoresidue-binding adaptor protein 14-3-3 to the I-BAR domain protein ITSp53 that plays an essential role in filopodia dynamics.

IRSp53 couples GTPase signaling with structural remodeling of the cytoskeleton and the membrane. In this role, IRSp53 is implicated in a number of normal development processes, but also in the pathology of for example cancer and neurological disorders. This fact is briefly mentioned in the introduction, maybe here (or later in the discussion) it would be helpful for the reader not accustomed to the role of IRSp53 to discuss the consequences of the negative influence of 14-3-3 on IRSp53. What might be the consequence of the here described regulation of 14-3-3 on IRSp53? What is the relevance of these findings for tumorigenesis, tumor progression, or metastasis? What does the community know about the possible effects in neurological disorders?

Back to the manuscript and the presented results, this study aimed at answering the following questions:

1) What are the phosphorylation sites in IRSp53 that mediate binding to 14-3-3 proteins?

2) What might be the protein kinases responsible for phosphorylating these 14-3-3 binding sites?

3) How do the multiple phosphorylation sites work together to convey 14-3-3 binding?

4) What is the stoichiometry of the interaction?

5) What is the effect of 14-3-3 binding on the overall conformation and activity state of IRSp53?

6) What is the relationship between 14-3-3 binding and the interactions of other partner proteins binding to IRSp53?

These questions were answered in the manuscript by a series of very elegant experiments. First the authors purified phosphorylated and 14-3-3 binding competent IRSp53 from HEK293 cells, using both a FLAG-tag and 14-3-3 affinity chromatography in a sequential manner to obtain pure and intact IRSp53, as can be judged from Figure 1c. (I wonder if the authors' did try to reconstitute this protein with heterologously in E. coli expressed 14-3-3 for co-crystallization, SAXS or cryo-EM…) For this purification it was helpful that the authors found out that previously reported 14-3-3 binding site in IRSp53 could be phosphorylated by AMPK, which can be induced by staving the cells prior to purifying the protein.

Next, further 14-3-3 interaction sites were predicted by in-silico methods and the phosphorylation status of these sites were tested using phosphoproteomics on purified IRSp53, revealing 5 sites were indeed phosphorylated in cells. These putative 14-3-3 binding sites were then synthesized as either mono- or di-phospho peptides and tested

via ITC for their ability to bind to 14-3-3. The advantage of ITC is that it not only delivers a Kd of the interaction but also the molar ratio and thermodynamic parameters, confirming the validity of 4 of the 5 predicted 14-3-3 binding sites, a result further corroborated by site specific mutation of these sites in full-length IRSp53 and 14-3-3 pulldowns from cells.

In the following, the expression of heterodimers of IRSp53 with one protomer no longer able to bind 14-3-3, resulted in the reduction of IRSp53 bound 14-3-3 by 50%, hinting at a binding stoichiometry of one IRSp53 monomer binding one 14-3-3 dimer, thus a 2:4 (IRSp53:14-3-3) complex. This result was strengthen by experiments using a FRET assay.

The next step in elucidating structural details of the 14-3-3/IRSp53 interaction was the elucidation of the crystal structures of 3 binding motifs in complex with 14-3-3 as singly phosphorylated peptides and two combinations (2,4 and 2,3) of these sites as doubly-phosphorylated synthetic peptides. Here, it is of importance that the simultaneous binding of two sites within one 14-3-3 dimer could be shown and that the structures of the single-site phosphorylation with 14-3-3 showed very similar binding architectures as the same sites in the doubly-phophorylated constructs. Maybe it would be a good idea to prepare a figure for the SI with a structural alignment of the 340, 360 and 366 sites between the mono- and the double-phosphopeptide structures reporting also the rsmd.

Finally, by employing a FRET sensor construct, the effect of 14-3-3 binding on the overall structure of IRSp53 and the influence of other partner proteins like Cdc42C andEps8 were analyzed.

All in all, this is a very important study reporting data of highest technical value that explain how the important protein IRSp53 is regulated by 14-3-3 proteins.

1. The only thing that might be added is a short discussion if and how these structural and mechanistic insights could be useful for example for potential drug discovery or chemical biology probe design.

   We appreciate the very positive comments of the reviewer. Honestly, we are not sure whether our results can directly help in drug discovery, but one possibility now highlighted in the Discussion (which was profoundly revised) is the use of our FRET sensor as a screening tool in drug discovery, both for IRSp53 as well as other 14-3-3 ligand (p-12, lines 18-25).

Minor remarks:

2. Page 10: '…we expressed in HEK293T cell a modified…' should be '…we expressed in HEK293T cells a modified…'
   Corrected (p-10, lines 24-25)

3. Page 10: '…this mutant fails to coimmunoprecipitation 14-3-3 from cells...' should be '…this mutant fails to coimmunoprecipitate 14-3-3 from cells…'

Corrected (p-10, line 27)

Reviewer #2 (Remarks to the Author):

This manuscript by Kast and Dominguez describes detailed studies of IRSp53 and 14-3-3 protein binding. The work reported in this manuscript is highly mechanistic and appears to be performed very well. I only have a few points of questions regarding this work. Overall, I think the work is very much straightforward and easy to understand.

Comment: I thought the discussion section could be more detailed in terms of discussing the proposed model, as opposed to talking about the pros and cons of various different online servers for protein-binding predictions. This part felt like it came out of nowhere.

We agree with the reviewer, and as a result completely rewrote the Discussion. The point about the performance of algorithms predicting 14-3-3-binding sites was shortened (p-13, lines 1-6). New paragraphs were added discussing: ***a***) the relationship of our findings to the general "gatekeeper" model of 14-3-3 function (p-13, lines 9-23), ***b***) kinases that could be implicated in the phosphorylation of IRSp53 at 14-3-3 binding sites (p-13, lines 25-34 & p-14, lines 1-12), ***c***) the potential use of our FRET sensor assay in the study of other 14-3-3 targets as well as a screening tool in drug discovery (p-12, lines 18-25), ***d***) the role of 14-3-3 on the regulation of the pIRSp53 interaction with membranes (page 12, line 13-15).

1. Dye-labeling is performed in a way to produce a mixture with variable labeling orientations. As such, it should be stated that experiments were performed using a single preparation of the labeled probes to make sure that assays were performed using a consistent/identical batch of probes.

   As suggested, we used the same batch of probes and labeled with the same ratio of donor/acceptor fluorophores. We now clarify this point in in the legend to Figure 5 (p-24, lines 11-12) and in the Methods (p-17, line 27).

2. Further increase in FRET, though small, was noted when Cdc42 was titrated into 14-3-3/pIRSP53fs, as in Fig.5c. Figure 5e shows a clear case of where there's no additional change in FRET upon titration of Eps8 (a complete flat line), but Fig. 5c shows a downward sloping curve as a function of increasing Cdc42 concentration. This needs to be explained. In the text, it simply says there was no effect from titrating in additional Cdc42 which is clearly not what the data suggests.

   We now clarify this observation in the text (p-9, lines 26-28)

3. In Figure 5g (right side blue colored plot), titration w/ Eps8 is shown in the figure panel, but the text states that this is titrated with 14-3-3 protein instead.

   Corrected in the Figure 5g.

4. Is Rac1 able to compete for activation identically to Cdc42? The main abstract/intro discusses Cdc42 and filopodia. Rac1-IRSP53 and signaling through WAVE-Arp2/3 should be discussed to give broader perspective.

   In previous work (Kast et al., NSMB 2014) we demonstrated that Rac1 does not directly bind to IRSp53 and, therefore, cannot compete with Cdc42. Rac1 probably acts indirectly on IRSp53. Therefore, we don't feel this needs to be re-discussed here, because: a) it would be repetitive and b) it is not the focus of the current work (regulation of pIRSp53 by 14-3-3 is the focus)

5. I recommend professional editorial service: writing is not clear in a number of places (does not flow). I had no idea what “servers” meant in the discussion, it just came out of the blue until I realized later that this is something about a web-based server platform… also some typos were noted.

   Indeed, ‘severs’ was not a clear term to use in this case. As stated above, this part of the Discussion was shortened and the term ‘algorithms’ is used instead. We also made other changes throughout the text (highlighted blue), and while a typo may still subsist, we believe the paper does not need a professional editorial service

Reviewer #3 (Remarks to the Author):

Comments for Kast et al
This paper describes interesting structure function relationship of the phosphorylation and the Cdc42 binding to IRSp53, a well-known I-BAR domain-containing protein for membrane protrusions. The AMPK induced phosphorylation of IRSp53 appeared to induce the binding of 14-3-3, and thereby inhibiting the IRSp53 by the clamp-like binding. The model is quite interesting, but I would like to point several issues that are unclear in the manuscript.

1. The responsible kinase is AMPK and some other kinases. The previous literatures said GSK3beta or some related kinase might phosphorylate IRSp53. The AMPK-induced phosphorylation and the suppression of the IRSp53 for the membrane binding looks to be reasonable because AMPK is activated upon serum starvation, which normally inactivates Cdc42. But how do the other kinases fit to the inhibition of IRSp53?

   This is an important but still unresolved question, given that only AMPK has been shown to directly phosphorylate IRSp53, and this is also consistent with our own results. Other kinases have been reported to have an indirect role on IRSp53’s

phosphorylation (GSK3β, LRRK2 and PKD). This issue is now addressed in the Discussion (p-13, lines 25-34 & p-14, lines 1-12).

2. The output of the phosphorylation-induced 14-3-3 binding appears to be the suppression of the membrane binding. The change in the membrane binding should be shown experimentally in the presence of 14-3-3.

   This is an important recommendation, and we are happy to report that these experiments were performed, resulting in a new section in the paper (*Binding of 14-3-3 reduces pIRSp53's association with membranes*) and a new Figure part (Fig. 6a). We performed these experiments in cells, which we believe is more relevant for our understanding of IRSp53's physiological activity. By fractionating fed and serum starved cells expressing either WT IRSp53-FLAG or M234-FLAG (a mutant that does not get phosphorylated and does not bind 14-3-3), we demonstrate that the fraction of IRSp53 bound to membranes markedly increases for the mutant. Furthermore, by analyzing fed and serum-starved cells (which leads to increased IRSp53 phosphorylation), we also show that starvation reduces the amount of WT IRSp53-FLAG bound to membranes, but not M234-FLAG.
   The new section appears in (p-11, lines 2-16). There is a mention in the Discussion (p-12, lines 13-16). In the Methods, there is a new section entitled *Membrane fractionation* (p-20 and 21). The legend to Figure 6 also changed (page 24-25). The new or edited text is highlighted blue.

3. The effect of 14-3-3 on cellular function of IRSp53, filopodia, might be better to be confirmed in your system.

   We agree – it is important to understand how the regulation of pIRSp53 by 14-3-3 affects cell behavior, and in particular filopodia formation and cell migration. While we do include a significant amount of cellular data in the current paper (Fig. 1, Fig 2f, Fig. 3a-b, Fig. 5f, Fig. 6a, Fig. S2), the analysis of cellular phenotypes resulting from this regulatory mechanism is part of another study, including a large amount of data in live cells. The corresponding manuscript is currently being prepared for submission.

4. Minor point: On page 2, BAIA2 should be BAIAP2.

   Corrected (p-2, line 13)

Reviewers' comments:

Reviewer #3 (Remarks to the Author):

Comments for Kast et al

The manuscript was improved because the author provided the data for membrane binding dependent on possible 14-3-3 binding by fractionation of cells into cytosolic and membrane fractions. However, the data do not have controls for membrane fractionation. Some marker proteins should be examined to demonstrate successful membrane fractionation for each step, such as mitochondria, plasma membrane, nuclei, and so on. Ideally the authors should purify the phosphorylated IRSp53 or make phosphorylated IRSp53 by in vitro kinase reaction, and then the 14-3-3-dependent membrane binding will be better to be examined using the in vitro reconstitution assay using purified proteins.

Response to Additional Comments from Reviewer-3

The manuscript was improved because the author provided the data for membrane binding dependent on possible 14-3-3 binding by fractionation of cells into cytosolic and membrane fractions. However, the data do not have controls for membrane fractionation. Some marker proteins should be examined to demonstrate successful membrane fractionation for each step, such as mitochondria, plasma membrane, nuclei, and so on. Ideally the authors should purify the phosphorylated IRSp53 or make phosphorylated IRSp53 by in vitro kinase reaction, and then the 14-3-3-dependent membrane binding will be better to be examined using the in vitro reconstitution assay using purified proteins.

In response to this request, we revised the analysis of membrane binding by IRSp53 as a function of 14-3-3 binding and cell starvation. We apologize for the time it took us to address this issue; we encountered many difficulties finding appropriate antibodies that could reliably detect canonical proteins in the plasma membrane. After, testing 11 different antibodies, we were able to find a reliable E-cadherin antibody that adds the requested membrane fraction control. In the new analysis, we have weighted the membrane fraction according to the abundance of E-cadherin and the cytosolic fraction according to the combined abundance of GAPDH and β-actin. We have made the necessary changes in the text and Methods (sections highlighted in blue) and provide a new Figure 6a and legend. We hope this new analysis addresses the additional concerns of the reviewer.

We believe the analysis in cells is more relevant than using *in vitro* purified proteins for several reasons: ***a***. As explained in the text, we do not know precisely what are the kinases that phosphorylate all the 14-3-3-binding sites of IRSp53, ***b***. *In* vitro phosphorylation often produces artefacts, since kinases may phosphorylate additional sites. ***c***. The lipid composition of membranes in cells is more relevant than *in vitro* reconstituted membranes, ***d***. We cannot test for the effect of starvation *in vitro* (as we did in cells).
